# Effects of Curcumin and Ferulic Acid on the Folding of Amyloid-β Peptide

**DOI:** 10.3390/molecules26092815

**Published:** 2021-05-10

**Authors:** Evdokiya Salamanova, Mariyana Atanasova, Ivan Dimitrov, Irini Doytchinova

**Affiliations:** Faculty of Pharmacy, Medical University of Sofia, 1000 Sofia, Bulgaria; esalamanova@ddg-pharmfac.net (E.S.); matanasova@pharmfac.mu-sofia.bg (M.A.); idimitrov@pharmfac.mu-sofia.bg (I.D.)

**Keywords:** curcumin, ferulic acid, amyloid-β peptide, molecular dynamics simulation, folding, secondary structure, solvent-accessible surface area, hydrogen bonding

## Abstract

The polyphenols curcumin (CU) and ferulic acid (FA) are able to inhibit the aggregation of amyloid-β (Aβ) peptide with different strengths. CU is a strong inhibitor while FA is a weaker one. In the present study, we examine the effects of CU and FA on the folding process of an Aβ monomer by 1 µs molecular dynamics (MD) simulations. We found that both inhibitors increase the helical propensity and decrease the non-helical propensity of Aβ peptide. They prevent the formation of a dense bulk core and shorten the average lifetime of intramolecular hydrogen bonds in Aβ. CU makes more and longer-lived hydrogen bonds, hydrophobic, π–π, and cation–π interactions with Aβ peptide than FA does, which is in a good agreement with the observed stronger inhibitory activity of CU on Aβ aggregation.

## 1. Introduction

Polyphenols are a group of natural compounds known for their numerous beneficial effects on different functions of the human body. In addition to their well-known antioxidant properties [1], new effects have recently been identified. Several studies have found that flavonoids and phenolic acids are able to reduce the risk of type 2 diabetes by inhibition of glucose absorption [2,3], stimulation of insulin secretion [4,5] and reduction of hepatic glucose output [6,7]. Similar mechanisms underline the antiobesity effects of polyphenols [8]. The antioxidant activity of polyphenols is associated with anti-inflammatory effects [9] and reduced risk of cardiovascular disease [10]. It has been found that polyphenol-rich plant extracts have anticoagulant properties [11] and anti-cancer effects [12,13]. Green tea polyphenols have digestion-improving activity by promoting the growth of probiotic bacteria and inhibiting the growth of pathogenic ones [14]. The polyphenols in grape juice [15], cocoa [16] and *Ginkgo biloba* [17] are able to improve brain activity and boost memory and concentration.

**Figure 1:**
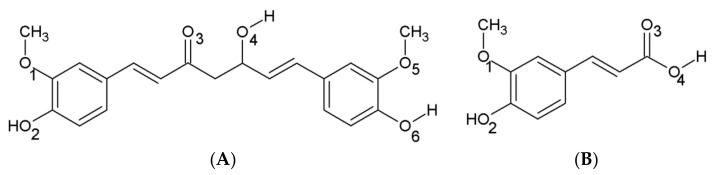
Chemical structures of the amyloid-beta (Aβ) inhibitors (**A**) curcumin (CU) and (**B**) ferulic acid (FA). The O-atoms in the molecules are numbered.

Curcumin (CU, Figure 1) is a natural polyphenol originating from the plant turmeric (*Curcuma longa*) [18]. Most of the beneficial effects of CU are due to its antioxidant [19] and anti-inflammatory properties [20]. CU is a free radical scavenger [19] and chain-breaking antioxidant [21]. It is an inhibitor of several enzymes involved in the generation of reactive oxygen species like lipoxygenase, cyclooxygenase and xanthine hydrogenase [22]. Based on its strong antioxidant properties, CU acts as an anti-inflammatory agent and prevents the development of many chronic diseases like neurodegeneration, cancer, osteoarthritis [18]. It was found that CU prevents the aggregation of amyloid-beta (Aβ) peptide [23] and tau proteins [24] by direct binding and prevention of their assemblage into neurotoxic species in the brain. The IC_50_ value of CU for in vitro inhibition of Aβ aggregation is 0.81 µM [23]. Moreover, CU is able to disintegrate preformed Aβ fibrils with EC_50_ of 1 µM [23].

Ferulic acid (FA, Figure 1) is a phenolic acid with antioxidant [25], hypotensive [26] and anti-inflammatory properties [27] found in various fruits and vegetables. It has been shown that FA inhibits aldose reductase, a key enzyme involved in the development of insulin resistance and metabolic syndrome [28]. Additionally, FA has neuroprotective properties like antidepressant [29,30,31], antinociceptive [32] and antiepileptic effects [33], protection from ischemia [34,35], Parkinson’s disease [36] and inflammation [37,38]. FA is a weak inhibitor of the Aβ aggregation as well with IC_50_ of 5.5 µM [39].

The effects of CU on the structure of Aβ oligomers and fibrils have been studied by molecular dynamics (MD) simulations. MD is a computational method for in silico mimicking the movement of a molecule in a given medium [40]. The effects of CU on the stability of dimers and protofibrils have been simulated and the mechanisms of interactions have been investigated at atomistic level [41,42,43,44,45,46,47]. Recently, we simulated the primary nucleation process of 12 Aβ peptides and studied the effects of CU and FA on the process [48]. We found that CU intercalates among the peptide chains, binds tightly to Aβ by hydrogen bonds, hydrophobic, π–π, and cation–π interactions and prevents the formation of a compact primary nucleus. The interactions of FA with Aβ are weak and short-living in accordance to the weaker inhibitory effect of FA on the Aβ aggregation.

Here, we examine by MD simulation the effect of CU on the secondary structure and folding of a single Aβ molecule and identify the interactions between the two molecules. The process of Aβ packing in saline is conducted for a duration of 1 µs (1000 ns) in the presence and absence of CU. For comparison, the same process for the same time and at the same conditions is conducted in the presence of the weaker inhibitor FA.

## 2. Models and Methods

### 2.1. Modeled Ligands and Systems

The structures of the two ligands CU (CID 969516) and FA (CID 445858) were downloaded from PubChem [49]. The CU was taken in tautomeric keto-enol form as it has been found that this form is able to prevent Aβ fibril formation [50,51]. The FA acid was simulated in anionic form as it is a weak acid with pK_a_ = 4.58. Structurally, FA mirrors the half-cutoff molecule of CU.

The point charges of CU and FA were derived as AM1-BCC charges using the AMBER module antechamber [52]. The topology of the Aβ peptide was built using the ff14SB force field. CU and FA were parameterized with GAFF atom type [53].

**Figure 2:**
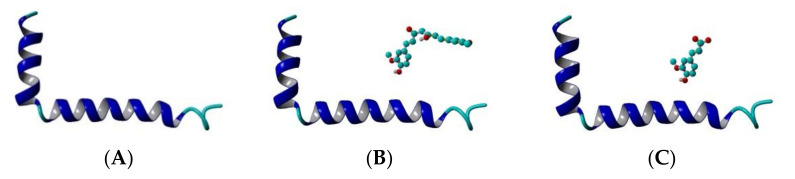
Initial topology of the systems modelled in the present study: (**A**) Aβ peptide, (**B**) Aβ peptide and CU, (**C**) Aβ peptide and FA. The peptide is given in cartoon, the ligand–in ball-and-stick. Structures are visualized by YASARA [55].

The Aβ_1-42_ monomer structure was obtained from PDB (pdb code 1IYT) [54]. Both ends were capped and all hydrogens were added. Three systems were arranged: Aβ peptide, Aβ peptide and CU, Aβ peptide and FA (Figure 2). The ligands were placed randomly near the peptide and the systems were solvated in a truncated octahedron water box using periodic boundary conditions (PBC) with TIP3P water explicit solvent molecules. Na+ and Cl- were added to neutralize the structures and achieve a salt concentration of 0.1 mol/L to mimic the physiological pH.

### 2.2. Molecular Dynamics Protocol

The MD simulations in the present study were performed using AMBER 18 [56]. As a first step in the MD protocol, the three systems were minimized for 10,000 steps using 10 kcal/molÅ^2^ harmonic restraints on solute heavy atoms and 10 Å cutoff for the non-bonded van der Waals and electrostatic interactions. The structures were heated to 300 K in a timestep of 2 fs with releasing the restraints to 3 kcal/molÅ^2^ for 100 ps. The next step was a system density equilibration for 100 ps under the same force constant on the heavy atoms. The restraints were released in 1 ns equilibration at constant temperature maintained with Langevin thermostat (300 K) and constant pressure using the Berendsen barostat (1 bar). The production run was held for 1 µs for any of the three systems. Frames were saved every 1 ns for a total of 1000 per trajectory.

The trajectories were analyzed by cpptraj V4.24.0 [57]. The following parameters were calculated: root mean square deviation (RMSD) accounts for changes in atomic coordinates of input frames to a reference frame (frame #1 by default); RMSF (root mean square fluctuations) accounts for atomic positional fluctuations; secondary structural propensities for residues (α-, 3_10_ and 3_14_ helices, parallel and anti-parallel β-sheets, turns and bends); solvent accessible surface area (SASA) accounts for the surface area in Å^2^ of atoms; hydrogen bond formation. The RMSD and RMSF were calculated for the backbone atoms of Aβ peptide. The calculation of secondary structure propensities was based on the DPSS method of Kabsch and Sander [58]. The SASA was calculated according to thr Weiser, Shenkin, and Still LCPO approximation method [59]. The electrostatic potentials on the SASA were calculated by Poisson-Boltzmann equation and visualized by PBEQ Solver [60]. The hydrogen bonds were calculated by applying simple geometric criteria for Acceptor-H-Donor distance cutoff of 3.0 Å and dihedral cutoff of 135°.

## 3. Results

**Figure 3:**
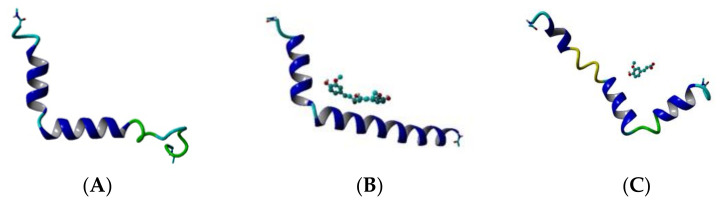

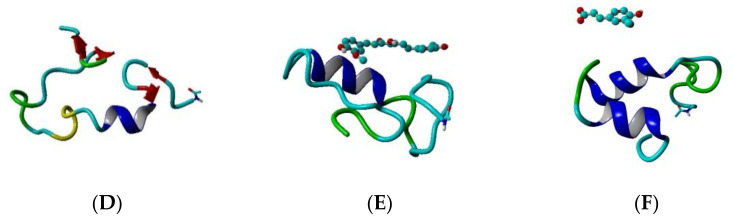
Topologies of the modelled systems at the beginning (frame #1) and at the end (frame #100,000) of the production phase: (**A**) Aβ peptide at frame #1, (**B**) Aβ peptide and CU at frame #1, (**C**) Aβ peptide and FA at frame #1, (**D**) Aβ peptide at frame #100,000, (**E**) Aβ peptide and CU at frame #100,000, (**F**) Aβ peptide and FA at frame #100,000. The peptide is given in cartoon, the ligand–in ball-and-stick.

The three systems–Aβ peptide, Aβ peptide and CU, Aβ peptide and FA–were solvated in water, energy-minimized, heated to 300 K, equilibrated and simulated for 1 µs (1000 ns) as described in the Models and Methods section. The topologies of the modelled systems before and after the production phase are given in Figure 3. The derived trajectories were analysed to assess the effects of CU and FA on the folding of Aβ peptide. 

### 3.1. Curcumin Stabilizes the Aβ Structure

**Figure 4:**
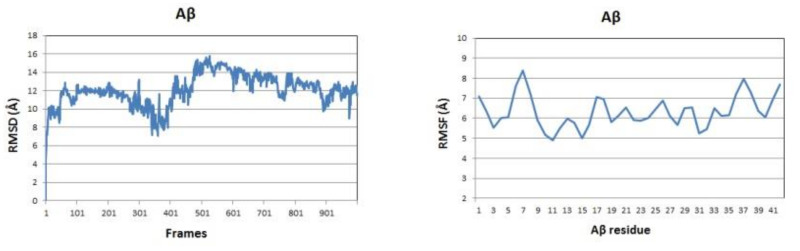

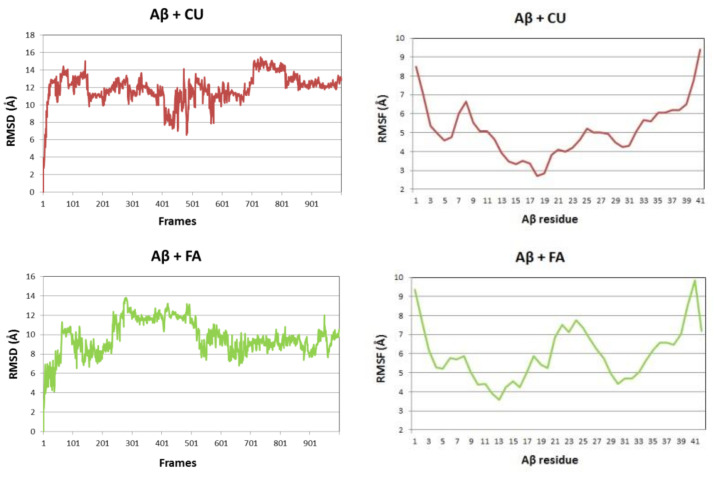
Aβ backbone RMSDs (**left** panels) and RMSFs per residue (**right** panels) of the modelled systems: Aβ peptide (blue), Aβ peptide and CU (red), Aβ peptide and FA (green).

The backbone RMSDs of the Aβ peptide in the three studied systems as a function of time are given in Figure 4 (left panels). Initially, the single Aβ molecule reaches equilibrium for 100 ns, stays stable for the next 200 ns, between 300 and 500 ns undergoes a distortion and after 500 ns it gradually stabilizes again. The difference between maximum and minimum Aβ backbone RMSDs over the last 100 frames of the simulation is 4.762 Å. In the presence of CU, the peptide is stabilized for the first 50 ns, stays stable for the next 300 ns, between 400 and 600 ns undergoes a distortion and after 700 ns is stable again till the end of simulation. The difference between maximum and minimum Aβ backbone RMSDs over the last 100 frames of the simulation is only 1.621 Å. In the presence of FA, the peptide distortions continue until 700 ns and then it stabilizes with difference between maximum and minimum backbone RMSDs over the last 100 frames of 4.383 Å. Obviously, the addition of CU to the solvated Aβ stabilizes the peptide structure. 

The backbone RMSF values per residue showed significant differences between the three simulated systems (Figure 4, right panels). The RMSF of the single Aβ peptide ranges from 4.90 to 8.38 Å with average value of 6.32 (±0.80) Å. The fluctuations are spread almost evenly along the sequence. In the presence of CU, the peptide structure is stabilized in the middle part between residues 9 and 32, while the both ends remain flanking. The RMSF of the peptide ranges from 2.70 to 9.39 Å with average value of 5.11 (±1.43) Å. FA has a weaker stabilizing effect focused mainly on the fragments 9–21 and 28–34 with RMSF values in the range 3.57–9.84 Å and average of 5.96 (±1.43) Å.

### 3.2. Curcumin and Ferulic Acid Increase the Helical Structure Propensity of Aβ Peptide

The propensities of secondary structures of the modelled systems are given as average values over the total 1000 frames (1000 ns) in Table 1. A-helix and parallel β-turn are the dominating structures in the single Aβ peptide, followed by anti-parallel β-turn. 3_10_ helices and bends are rare. In the presence of CU, the propensity of α-helix increases, the parallel β-turn decreases, the anti-parallel β-turn almost disappears, bends increase slightly and 3_10_ helix does not change. Similar changes were observed in the presence of FA.

The propensities of α- and 3_10_-helices were summed and presented as helical structure propensity, while the sum of propensities of parallel and anti-parallel β-turns and bends gives the non-helical propensity (Table 1). Both CU and FA increase the helical propensity and decrease the non-helical propensity of Aβ peptide.

**Figure 5:**
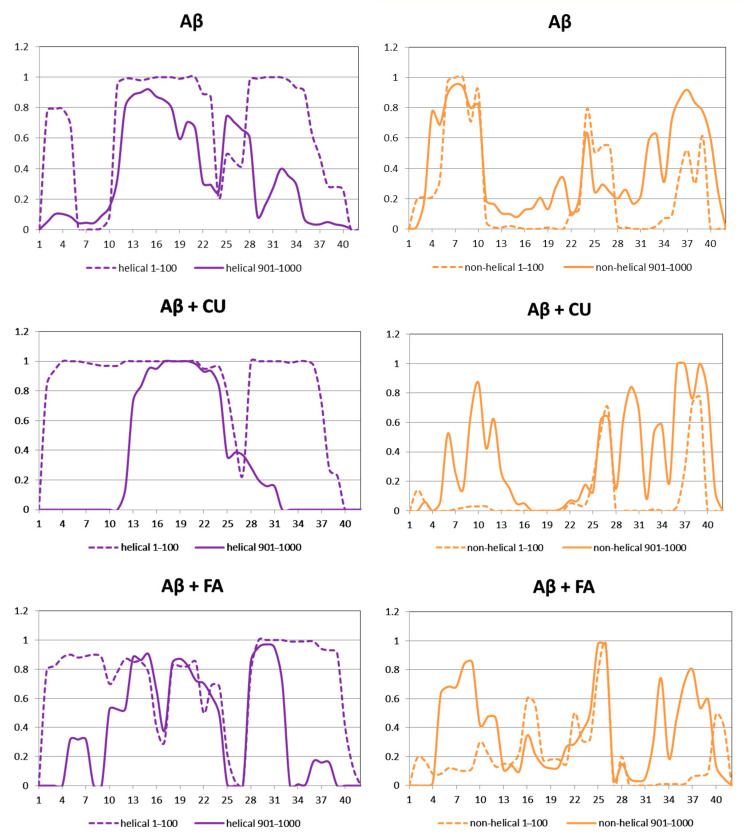
Propensities of helical and non-helical structures of the modelled systems averaged over the initial 100 ns (dash lines) and the final 100 ns (solid lines) of the production phase.

The helical and non-helical propensities averaged over the initial 100 ns and over the final 100 ns of the production phase of the MD simulation are presented along the peptide sequence in Figure 5. Initially, three helices exist in the structure of Aβ peptide (Figure 3A). They include residues 2–5, 11–23 and 28–36. At the end of simulation, the first helix has disappeared, the second has shrinnked between positions 12–21 and the third has split into two shorter helices between position 25–28 and 31–34. Between the helices non-helical domains are spread. Initially, they involve positions 6–10, 24–27 and 37–39. At the final, the non-helical structures are localized at the two peptide ends including positions 4–10 and 32–40. Two anti-parallel β-turns are formed here as is shown in Figure 3D.

During the initial 100 frames (100 ns) of the production phase in the presence of CU, two well-defined α-helices cover almost the whole structure of Aβ peptide and two short non-helical domains exist including residues 26, 27, 38 and 39 (Figure 3B). At the end of simulation, an α-helix exists only between positions 13–24, while the remaining part of the peptide contains non-helical structures (Figure 3E).

In the presence of FA, the initial structure of Aβ consists of three helices and three non-helical structures (Figure 3C). At the final 100 frames, the peptide still contains three shorter helical structures but at the end of simulation the last one is disordered (Figure 3F).

### 3.3. Curcumin and Ferulic Acid Increase the Solvent-Accessible Surface Area (SASA)

SASA accounts for the density of packing of the peptide molecule in saline. The average SASA over the production trajectory of a single Aβ molecule is 3237 Å^2^. In the presence of CU, the average SASA of Aβ increases to 3586 Å^2^. In the presence of FA, it reaches 3679 Å^2^. Both ligands prevent the formation of a dense bulk core and disfavor the growth of packing.

**Figure 6:**
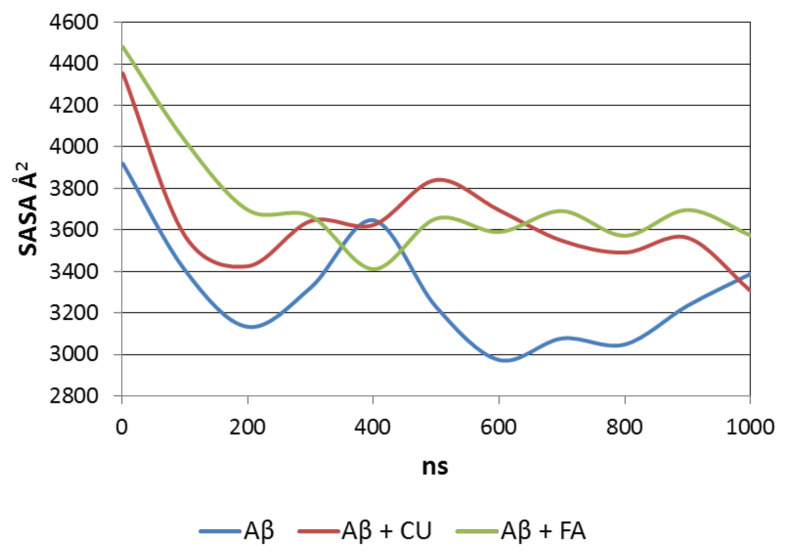
SASA averaged over every 100 ns of the production trajectory of Aβ peptide in the three modelled systems.

The level of packing of the Aβ peptide is visualized by SASAs averaged over every 100 ns for the three analysed systems (Figure 6). The single Aβ molecule formes in a two-step manner a compact nucleus which slightly relaxes at the end of simulation. In the precense of CU, the peptide initially shrinks, then relaxes, followed by small decrease and increase in SASA. Similar behaivior has Aβ in the presence of FA.

**Figure 7:**
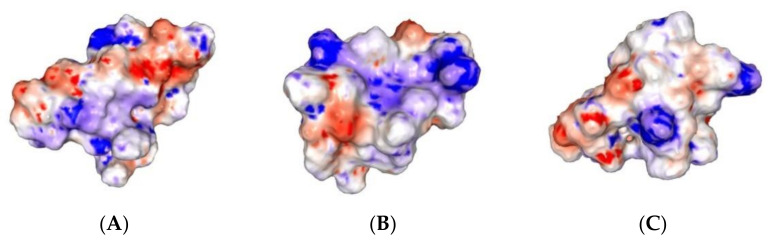
3D electrostatic potential map on SASA visualized by PBEQ Solver. (**A**) Aβ peptide; (**B**) Aβ peptide and CU; (**C**) Aβ peptide and FA. Areas with negative potential (−2 kcal/(mol.e)) are given in red, areas with positive potential (+2 kcal/(mol.e))–in blue, areas with neutral potential–in white.

The electrostatic potentials on the SASA calculated by Poisson-Boltzmann equation and visualized by PBEQ Solver showed that the compact nucleus of Aβ peptide formed in the three systems consists of a hydrophobic core and a hydrophilic polar surface (Figure 7). As an entropy-driven process, the packing of Aβ in saline forms nuclei of different shapes and surface arrangements.

### 3.4. Curcumin Forms More and Longer-Lived Hydrogen Bonds with Aβ Peptide

During the 1 µs simulation, 444 intramolecular hydrogen bonds in total were recorded between the Aβ residues with average lifetime of 30.53 ns (Table 2). Among the longest-lived hydrogen bonds are those between Gln15 and Phe19, Glu11 and Gln15, and Val24 and Lys28 with lifetimes >400 ns.

**Figure 8:**
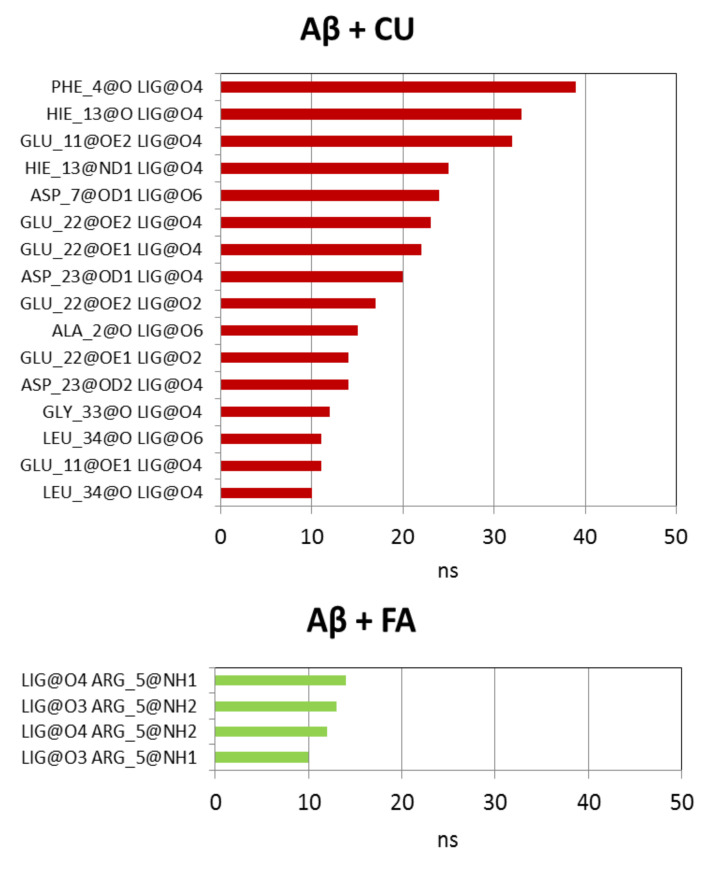
Intermolecular hydrogen bonds between Aβ peptide and ligands CU and FA with lifetime ≥10 ns.

In the presence of CU, the intramolecular hydrogen bonds in Aβ are preserved as a number but decreased as an average lifetime to 26.59 ns. Ninety four short-living intermolecular hydrogen bonds are formed between Aβ and CU with average lifetime of 5.46 ns. In 65% of these bonds, CU acts as a donor through the phenolic OH groups and the enol group. The longest-lived hydrogen bonds between Aβ and CU are given in Figure 8. As corresponding acceptors in these bonds act the backbone O-atoms of Phe4, His13, Ala2, Gly33 and Leu34, the side-chain O-atoms of Glu11, Asp7, Glu22 and Asp23, the imidazole N-atom (Nδ) of His13.

The presence of FA in the vicinity of Aβ increases the number of intramolecular hydrogen bonds but also descreases the average lifetime to 25.91 ns (Table 2). Only 55 hydrogen bonds between Aβ and FA were detected during the simulation with ultra-short average lifetime of 2.67 ns. In 75% of them FA acts as an acceptor mainly through the carboxy O-atoms (Figure 8). The longest-lived hydrogen bonds involve the guanidino group of Arg5.

## 4. Discussion

The single monomer Aβ peptide is a water-soluble non-toxic molecule. In an apolar microevironment, it exists as two α-helices connected by a flexible kink [54] as is shown in Figure 2A. In water, the peptide adopts a collapsed compact coil structure [61]. This structure is meta-stable and is able to be easily arranged into oligomers, protofibrils and fibrils containing predominantly intermolecular β-sheet structures which are water insoluble and neurotoxic [62]. Thus, the prevention of nascent Aβ monomer from misfolding and conversion to toxic conformers can serve as a rational therapeutic goal.

It was proven by in vitro studies that the polyphenol CU can block the formation of Aβ oligomers [23]. Additionally, CU is able to cross the blood-brain barrier and to bind to and disintegrate preformed amyloid plaques in mice brains [23]. However, the submolecular mechanisms of the interactions between CU and Aβ and the effects of CU on the process of folding of a single Aβ molecule are still unclear. Even more unclear are the effects of the phenolic acid FA on the folding of Aβ. FA could be considered as a half-cutoff molecule of CU with almost 7-fold weaker inhibitory activity on Aβ aggregation [39].

The aim of the present study is to analyze the effects of CU and FA on the folding process of a single Aβ peptide molecule in a 1:1 ratio in saline. CU is a strong inhibitor of Aβ aggregation, while FA is a weak inhibitor. Additionally, CU is a neutral molecule, while FA is an anion at pH 7.4. The two molecules were positioned randomly in a close proximity to Aβ peptide and the interactions between them were conducted in an octahedron box of water molecules and NaCl for a time of 1 µs. The movements and interactions were described quantitatively by parameters calculated over the trajectories. For comparison, the movement of a single Aβ peptide in the same box for the same time was also conducted.

The quantitative parameters derived from the MD simulation reveal several similarities and dissimilarities in the effects of the two inhibitors on the folding of Aβ. CU has a stronger stabilizing effect on Aβ structure than FA. Stabilization is achieved for 50 ns. Except for both ends, the whole main structure of Aβ is stable in the presence of CU. In the presence of FA, two peptide fragments remain stable: residues 9–21 and 28–34. The middle part of the peptide (residues 22–27) and both ends are rather flexible.

Both ligands have similar effects on the secondary structure of Aβ. They increase the propensity of α-helices and decrease the propensities of β-turns. In the presence of CU, the helical structure involves the middle peptide part (residues 13–24). In the presence of FA, this helical structure splits into two short helices including residues 10–16 and 18–23 with a bend at position 17. In the absence of ligands, the helical structure of Aβ gradually decreases and converts either into random coil or into a hairpin β-strand. At the end of simulation, two β-strands are formed (residues 4–10 and 32–40). The formation of such regions of β-turns is considered as the first step, from which the subsequent assembly of misfolded peptides proceeds [63].

Both ligands increase the SASA of Aβ peptide with 11% (CU) and 14% (FA). The Aβ nuclei formed in the absence or presence of ligands are similar–they consist of hydrophobic core and hydrophilic polar surface of different shape and arrangement. The number of intramolecular hydrogen bonds in Aβ peptide is not affected by the ligands but both shorten the average lifetimes of the bonds. CU binds to Aβ by 94 hydrogen bonds with average lifetime of 5.46 ns. In most of them, CU acts as a hydrogen-bond donor mainly through the phenolic OH and enol groups. The longest-lived bonds exist between 30 and 40 ns. On the contrary, FA forms half of the CU bonds with shorter average lifetime of only 2.67 ns. In these bonds, FA acts dominantly as a hydrogen-bond acceptor through the carboxy O-atoms. The longest-living hydrogen bonds of FA last up to 15 ns.

**Figure 9:**
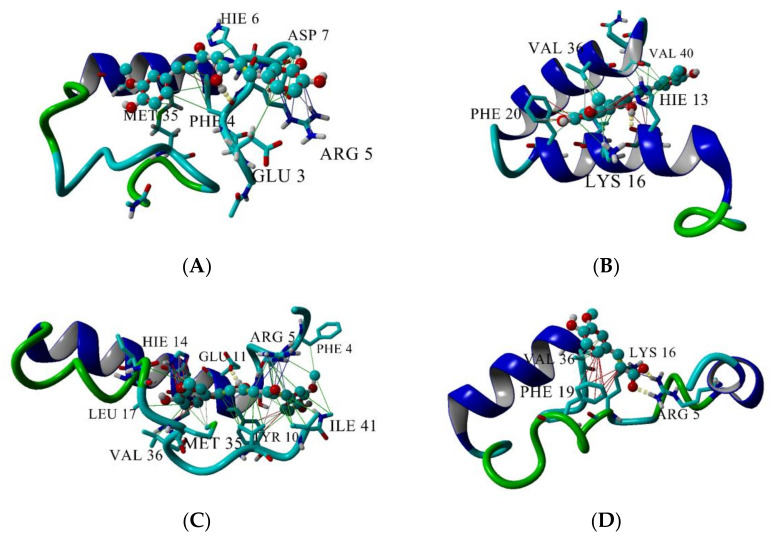
Interactions between CU and Aβ peptide (**A**–**C**) and between FA and Aβ peptide (**D**). The peptide is given in cartoon, the ligand–in ball-and-stick. Hydrogen bonds are given by yellow dashes, hydrophobic interactions–by green lines, π-π interactions–by red lines and cation-π interaction–by blue lines. Interactions are visualized by YASARA [55].

The top three longest-lived interactions of CU and FA with Aβ peptide are given in Figure 9. CU makes hydrogen bonds with Phe4 (Figure 9A, yellow dashes), His13 (Figure 9B) and Glu11 (Figure 9C). FA binds bidentately to Arg5 (Figure 9D) making two parallel hydrogen bonds between the carboxy O-atoms of FA and the N-atoms of guanidino group of Arg5. Apart from the hydrogen bonds, the two ligands are involved in a variety of intermolecular interactions with the peptide. CU makes hydrophobic contacts with Glu3, Phe4, Arg5, His6, Asp7, Met35 (Figure 9A, green lines), with His13, Lys16, Phe20, Val36, Val40 (Figure 9B), Tyr10, Leu17, Ile41 (Figure 9C). Additionally, CU is involved in π-π interactions with Phe4 (Figure 9A, red lines), His13 and Phe20 (Figure 9B), Tyr10 and His14 (Figure 9C) and in cation-π interactions with Arg5 (Figure 9A,C, blue lines) and Lys16 (Figure 9B).

In comparison with CU, FA makes fewer interactions with the Aβ peptide (Figure 9D). Apart from the bidentate hydrogen bond, FA interacts hydrophobically with Lys16 and Val36, participates in π-π stacking with Phe19 and cation-π bond with Arg5.

## 5. Conclusions

In conclusion, the polyphenols CU and FA affect the folding of Aβ peptide by increasing the helical and decreasing the non-helical propensities. CU forms numerous long-living hydrogen bonds, hydrophobic, π-π and cation-π interactions with the peptide, while the interactions of FA are fewer and short-living. These discrepancies in the interactions with Aβ are in a good agreement with the observed stronger inhibitory activity of CU on Aβ aggregation and the weaker activity of FA.

## Figures and Tables

**Table 1:** Propensities of secondary structures of the modelled systems given as average values over the total 1000 ns (1 µs). Helical propensity is the sum of α- and 3_10_- helices. Non-helical propensity summarizes parallel and anti-parallel β-turns and bending structures.

Secondary Structure	Aβ	Aβ + CU	Aβ + FA
Alpha helix	0.275	0.355	0.397
3_10_ helix	0.067	0.059	0.069
Anti-parallel β-turn	0.111	0.019	0.009
Parallel β-turn	0.211	0.192	0.205
Bend	0.081	0.109	0.090
Helical	0.342	0.414	0.466
Non-helical	0.403	0.320	0.304

**Table 2:** Number, percentage and lifetime of intramolecular and intermolecular hydrogen bonds, calculated over 1000 ns.

Hydrogen Bonds	Aβ	Aβ + CU	Aβ + FA
Total	444	535	533
Intramolecular	444 (100%)	441 (82%)	478 (90%)
Average lifetime (ns)	30.53	26.59	25.91
Intermolecular	-	94 (18%)	55 (10%)
Average lifetime (ns)	-	5.46	2.67
Ligand is a donor	-	61 (65%)	14 (25%)
Ligand in an acceptor	-	33 (35%)	41 (75%)

## Data Availability

N/A.

## Sample Availability

Samples of the compounds are not available from the authors.
